# Evaluation of the Clinical, Child, and Parental Satisfaction with Zirconia Crowns in Maxillary Primary Incisors: A Systematic Review

**DOI:** 10.1155/2021/7877728

**Published:** 2021-07-05

**Authors:** Mohammad Hassan Hamrah, Saeedeh Mokhtari, Zahra Hosseini, Maryam Khosrozadeh, Sepideh Hosseini, Elaha Somaya Ghafary, Mohammad Hussain Hamrah, Narges tavana

**Affiliations:** ^1^Department of Pediatric Dentistry, School of Dentistry, Tehran University of Medical Sciences, Tehran, Iran; ^2^Department of Pediatric Dentistry, Faculty of Dentistry, Kermanshah University of Medical Sciences, Kermanshah, Iran; ^3^Department of Periodontics, School of Dentistry, Kabul University of Medical Sciences, Kabul, Afghanistan; ^4^Andkhoy Curative Clinic, Andkhoy, Afghanistan

## Abstract

**Background:**

With the increasing demand for aesthetics in children and their parents, the treatment of decayed primary anterior teeth is one of the biggest challenges in pediatric dentistry. Zirconia crowns have provided a treatment alternative to address the aesthetic concerns and ease of placement of extracoronal restorations on primary anterior teeth.

**Methods:**

The electronic databases including PubMed, Scopus, Google Scholar, and Embase were searched on articles published between January 2010 and January 2021. Studies relating to evaluating the clinical success and satisfaction of both parents and children about zirconia crowns in maxillary primary incisors were reviewed.

**Results:**

Nine studies met the criteria for final inclusion. Findings from these studies showed that parental and child satisfaction with zirconia crowns is high with clinically acceptable restorations in the maxillary primary incisors.

**Conclusion:**

Parental and child satisfaction with zirconia crowns is high with clinically acceptable restorations in the maxillary primary incisors. In addition, larger sample sizes and longer follow-ups are required in future studies.

## 1. Introduction

Early childhood caries (ECC) is still one of the most common chronic diseases in children worldwide [1]. ECC does not only affect children's oral health but also the general health [2]. In addition, one of the major effects of ECC is the labial surface of upper anterior teeth, which results in the visibility of these carious lesions. Anterior teeth are mostly visible when eating, smiling, and speaking. Thus, visible anterior teeth have the greatest aesthetic value to individuals [1, 2].

Today, aesthetic dentistry is an essential component of modern dental practice. However, the knowledge of children's aesthetic perception is relevant to dentists and their parents , but children also have dental aesthetic perception of others of the same age [3, 4]. The aesthetic of the primary anterior teeth with ECC has been a major challenge for pediatric dentists. However, the requests of their parents have improved aesthetic solutions [5]. Furthermore, the management of decayed primary anterior teeth is particularly challenging for dentists due to behavioral management difficulties in young children. During the treatment of children, the dentist must have sufficient skills in the treatment plan, type of materials, and behavioral management, but these are not enough. Parental consent is also required [6]. The demand for beautiful smiles is growing among children as much as adults. A child's appearance is often associated with social acceptance, quality of life, and psychological and physiological development [7].

Today, we have a large number of solutions available for aesthetic problems in pediatric dentistry including full-coverage crowns for primary anterior teeth, composite strip crowns, preveneered stainless steel crowns (SSCs), and prefabricated primary zirconia crowns. Resin composite strip crowns are less retentive due to their high technical sensitivity [8]. Preveneered stainless steel crowns show a higher incidence of facial veneer fractures [9]. Zirconia crowns have high retention [10]. They have been used for more than two decades in permanent teeth with high acceptability and excellent mechanical properties [11]. In pediatric dentistry, EZ-Pedo introduced the first zirconia crowns in 2008, and since then, other companies have produced various zirconia crowns [12]. Zirconia crowns are retentive and gingival friendly but cause nonsignificant abrasion of opposing teeth [10]. They are biocompatible providing good marginal integrity, gingival health, and aesthetic [13]. Zirconia has a special ability to prevent crack propagation by transforming from one crystalline phase to another [13]. Zirconia crowns require a passive fit on placement; thus, they have a more open margin than SSC crowns, and their retention relies solely on the cement [11]. However, the zirconia crowns differ in the cement recommended by their manufacturers for their cementation. Traditional glass-ionomer cement, such as Ketac Cem, is recommended for EZCrowns. In contrast, BioCem cement is recommended for cementation of NuSmile® [14]. The aim of this study is to systematically review scientific evidence relating to evaluating the clinical success and parental satisfaction about zirconia crowns in maxillary primary incisors.

## 2. Materials and Methods

This systematic review was conducted following the Preferred Reporting Items for Systematic Reviews and Meta-Analyses (PRISMA) guidelines [15]; a specific question was constructed according to the PICO (population, intervention, comparison, and outcome) [16]. Our working hypothesis is the clinical success and parental satisfaction about zirconia crowns in maxillary primary incisors.

PICO:

- Population: maxillary primary incisors
- Intervention: zirconia crowns
- Comparison: clinical success and child and parental satisfaction outcomes of zirconia crowns
- Outcome: gingival health, tooth preparation, and survival rate

### 2.1. Search

The Medline (through PubMed), Scopus, Google Scholar, and Embase databases were explored through advanced searches and databases for articles published between January 2010 and January 2021 to perform a literature search on studies which investigated the parental and child satisfaction and clinical success of zirconia crowns of primary incisors. These databases were searched for articles published in the English language. The search keywords were (Pediatric) AND (zirconia Anterior Crowns) OR (zirconia) AND (anterior) OR (primary incisors) AND (Parental) AND (child). All records electronically identified were independently assessed by two authors according to their titles, abstracts, and/or keywords, and the full texts of all reports considered potentially relevant were obtained. Article types such as reviews, letters, and conference proceedings were excluded. Afterwards, articles from the initial search were screened for duplicates (using EndNote software version 8), followed by screening their titles and abstracts for conformity to the eligibility criteria. Furthermore, references of retained articles were manually screened for possible inclusion of relevant studies.

### 2.2. Study Selection

The obtained articles were independently subjected to clear inclusion and exclusion criteria by two authors (MHH and MK).

Inclusion criteria for the studies were as follows:

- Clinical studies with full texts
- Case report studies involving ZC primary incisors

Exclusion criteria for the studies were as follows:

- Conference abstracts, review articles, letters, editorials, unpublished data, articles without full texts, and non-English articles
- Studies which did not assess the efficacy of zirconia in primary incisors
- Follow-up less than 6 months
- Studies in which their full texts were not available

### 2.3. Sequential Search Strategy

Firstly, all retrieved articles from electronic as well as manual searches were entered into Endnote software (version X8, Thomson Reuters, New York, USA). Thereafter, duplicates were removed. Afterwards, two authors (MHH and MK) independently reviewed the titles and abstracts of the retrieved studies for eligibility. Studies were then selected based on the predetermined inclusion and exclusion criteria. For any disagreements concerning the inclusion of studies, all authors agreed on a consensus based on factual evidence.

### 2.4. Data Extraction

The data were extracted from the studies according to the aim of the systematic review by two authors (SH and MHH) independently and were arranged in the following fields: general information (name of the author and year of publication), country, study type, sample description, follow-up, children's age, pulp therapy, cement, zirconia crown brand, and main outcomes. Furthermore, they were summarized and presented in tables.

## 3. Results

**Figure 1:**
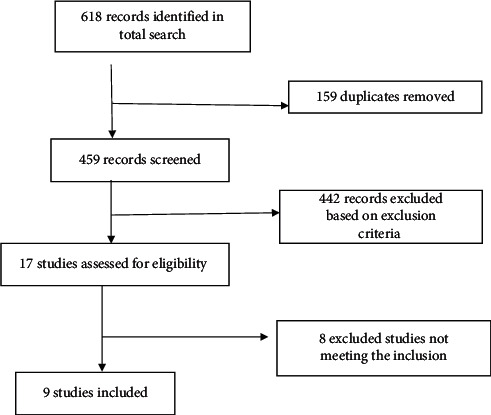
The selection process of the studies included in the systematic review.

The literature search yielded a total of 618 articles (Figure 1), from which 159 duplicate references were removed. The remaining 459 studies were imported into the EndNote library for further review. From these, 442 studies were excluded based on the inclusion and exclusion criteria. The remaining 17 articles were selected for a review of their full texts, after which 8 studies were screened out. The summary of the data showing the characteristics of included studies is presented in Table 1.

The screening process resulted in a total of 9 articles that were included in the present systematic review (Table 1). Of these, 3 were randomized controlled clinical trials, 3 were prospective cohort clinical investigations, 1 was a cross-sectional study, and 2 were case reports. The included studies showed that parental and child satisfaction with zirconia crowns is high with clinically acceptable restorations in the primary maxillary anterior dentition. All the participants of included studies had received pulp therapy under general anesthesia or sedation.

Studies comparing parental and child satisfaction with clinical success were conducted using three different tooth-colored anterior crowns which showed that parents and children had the highest satisfaction with zirconia crowns, followed by strip crowns and preveneered SSCs in primary anterior maxillary teeth. Zirconia crowns were found to have better aesthetics, retention, and gingival health at their follow-up [20, 22, 24]. Studies by Yanover et al. and Salama showed that zirconia crowns offer a better aesthetic and are a highly acceptable and restorative option for primary maxillary anterior teeth, as shown by 100% retentiveness, color match, absence of gingival irritation, and 94.7% cosmetic appearance with 100% very satisfied rating in the overall parental and children satisfaction [13, 20].

The case reports showed that zirconia crowns offer high-end aesthetics, superior durability, and easy placement compared to composite restorations and strip crowns. Therefore, they can be considered as a method of aesthetic rehabilitation in pediatric patients [17, 19].

One study compared prefabricated primary zirconia with resin composite strip crowns on primary maxillary central and lateral incisors with regard to gingival health, plaque accumulation, recurrent caries, restoration failure, and opposing tooth wear over a period of 3, 6, and 12 months. Zirconia crowns showed significantly less gingival bleeding, better gingival health, and plaque accumulation, as well as less loss of material [19]. One cross-sectional study evaluated the clinical success of parental satisfaction with anterior pediatric zirconia crowns for retention, gingival health, color match, contour, marginal integrity, and opposing tooth wear [23]. Parental satisfaction regarding the aesthetics of the crowns and parental perception of the impact of treatment on the child's appearance and oral health were evaluated using a questionnaire. Their results showed that the parents reported high satisfaction with the color, size, and shape of the crowns. Moreover, a majority of parents reported that crowns improved the appearance and oral health of their child [6, 18].

One study conducted by El Shahawy and O'Connell showed a simple reliable technique for restoring severely mutilated primary anterior teeth. A rigid glass-ionomer post was created over which zirconia crowns can be fitted to achieve a long-term stable aesthetic restoration for primary anterior teeth. The use of zirconia crowns offered superior aesthetic, durable restorations with remarkable gingival response up to 24 months [21].

## 4. Discussion

To our knowledge, this is the first study to systematically review the clinical evaluation and parental and child satisfaction with zirconia crowns in maxillary primary incisors. Our findings from the reviewed studies showed that parental satisfaction with zirconia crowns is high with clinically acceptable restorations in the maxillary primary incisors. The aesthetic of the primary anterior teeth with ECC has been a major challenge for pediatric dentists. However, the request of their parents has improved aesthetic solutions [6]. Recently, zirconia crowns have been introduced for primary anterior teeth [25]. The most apparent advantage of zirconia crowns is their excellent aesthetics, better gingival health, and plaque accumulation compared to SSCs, polycarbonate crowns, preveneered SSCs, and bonded resin strip [19]. In the studies conducted by Salami et al. [10], anterior zirconia crowns (43 crowns in 13 children) were followed after placement for clinical success over six months and 12 months, respectively, and had high retention rates as well as high parental satisfaction [10]. Holsinger et al. [6] followed placement of anterior prefabricated zirconia crowns (57 crowns in 18 children) after an average of nearly 21 months. The authors found high retention rates as well as high satisfaction from parents [6].

Zirconia crowns are less technique sensitive and more moisture tolerant. However, the potential disadvantages of the zirconia restoration are the inability to crimp the crown for mechanical retention, the inability to change its color, the limited ability to trim the crown or alter its shape, and the need for more tooth reduction than a traditional preformed metal crown [26]. Some drawbacks which limit the use of zirconia crowns are that it requires significantly more time to prepare the tooth for fitting the crown. Bleeding from the gum, due to anxiety or inflammation, may hinder the setting of the cement used to bond the zirconia crown to the tooth. With crying or inability to sit still and fully cooperate for the procedure, an SSC would be preferable since the preparation of the tooth and fitting an SSC take much less time, but with the latest innovations, manufacturers are trying to minimize these factors. Ez-Pedo has introduced Zir-Lock ultra, mechanical undercuts to increase crown retention. Another point to consider is that zirconia crowns not contaminated with blood or saliva have better adhesion to cement, and to solve this problem, NuSmile came up with the try-in pink crown [27]. After tooth preparation and size selection, gingival bleeding was controlled, and the teeth were rinsed and dried, followed by crown cementation according to the manufacturer's directions [13].

Zirconia crowns have been used successfully as clinical advantages were extended to pediatric dentistry. Zirconia crowns have gained popularity among pediatric dentists and have shown high parental and child satisfactory ratings compared to full-coverage crowns [12]. Thus, studies comparing zirconia crowns to resin composite strip crowns on primary maxillary incisors reported that zirconia crowns showed less gingival bleeding, better gingival health, and plaque accumulation. Thus, in one study with follow-ups of 12 and 24 months, severely decayed primary maxillary incisors treated with glass-ionomer posts and ZCs showed high success rate with overall survival of 95.3% at 12 months and 80.2% after 24 months [21]. A study which assessed the anterior primary crowns for 131 patients (aged between 24.8 and 2.2 months) reported that zirconia crowns showed improved overall marginal integrity, gingival health, and aesthetics [13].

It is important to understand that aesthetic harmony can lead to better psychological health and higher self-assurance. It improves peer relationships and strengthens self-confidence in a growing child. Zirconia crowns offer high-end aesthetics, superior durability, and easy placement compared to composite restorations and strip crowns. Therefore, they can be considered as a method of aesthetic rehabilitation in pediatric patients [17].

Despite the limited number of published clinical trials, the available studies showed that parental and child satisfaction with zirconia crowns is high with clinically acceptable restorations in the maxillary primary incisors [12, 13, 21]. However, larger sample sizes and longer follow-ups are required in future studies.

## 5. Conclusion

Findings from the reviewed studies have shown that parental and child satisfaction with zirconia crowns is high with clinically acceptable restorations in the maxillary primary incisors. However, there still remains a lot of work to be done in ensuring their full clinical translation. Further research studies are necessary, including in vitro, in vivo, and clinical studies with larger sample sizes and longer follow-ups.

## Figures and Tables

**Table 1:** Summary of main outcomes about clinical success and parental satisfaction about zirconia crowns in maxillary primary incisors.

First author and year	Country	Study type	Sample description	Follow-up	Children age	Pulp therapy	Cement	Zirconia crown brand	Main outcomes
Yanover et al., 2020 [13]	Canada	Retrospective cohort	131 ZCs, 36 children	6 to 33.8 months	24.8–62.2 months	Pulpotomy	Glass ionomer	EZCrowns (Sprig) NuSmile Cheng	ZC comprises a satisfactory treatment option for carious primary maxillary incisors, presenting good overall marginal integrity, gingival health, and aesthetics.

Banerjee et al., 2020 [17]	India	Case report	4 ZCs	6 months	4 years	Pulpotomy	Glass ionomer	—	Zirconia crowns offer high-end aesthetics, superior durability, and easy placement compared to composite restorations and strip crowns, so they can be considered as a method of aesthetic rehabilitation in child patients.

Chao, 2020 [18]	USA	Retrospective cohort	26 ZCs	12, 24, and 36 months	Average age: 40 months	Pulpotomy, pulpectomy	BioCem	NuSmile	Zirconia crowns demonstrated lower survival probabilities over 36 months compared to stainless steel crowns and resin strip crowns.

Ashima et al., 2014 [19]	India	Case report	4 ZCs	30 months	4 years	Pulpectomy	Light-cure resin cement (RelyX/3M ESPE)	ZIRKIZ, HASS Corp., Korea	The crowns have demonstrated good retention and aesthetic results.

Holsinger et al., 2016 [6]	UK	Cross-sectional	57 ZCs, 18 children	6 to 37 months	Average age: 20.8 months	Pulpotomy	Glass ionomer	EZ-Pedo	Zirconia crowns are clinically acceptable restorations in the primary maxillary anterior dentition. Parental satisfaction with zirconia crowns was high.

Salama et al., 2018 [20]	Egypt	Retrospective cohort	40 ZCs, 32 children	12 months	2–6 years	—	Glass ionomer	NuSmile	Zirconia crowns showed 100% retentiveness, color match, absence of gingival irritation, and 94.7% cosmetic appearance with 100% overall parental satisfaction rated as very satisfied.

El Shahawy and O'Connell, 2016 [21]	Egypt	Randomized controlled clinical trial	86 ZCs, 23 children	24 months	2–5 years	Pulpectomy	Glass ionomer	NuSmile	Glass ionomer-retained zirconia crown offers superior aesthetics and a durable restoration with remarkable gingival integration for the treatment of severely mutilated primary anterior teeth.

Gill et al., 2019 [22]	USA	Randomized controlled clinical trial	40 ZCs	12 months	3.4 years	Pulpectomy	Glass ionomer	NuSmile	Parental aesthetic satisfaction was highest for NuSmile ZCs.

Alaki et al., 2020 [23]	Saudi Arabia	Randomized controlled clinical trial	60 ZCs, 32 children	12 months	3 to 5.5 years	—	Light-cure resin cement (NuSmile BioCem)	NuSmile	Zirconia crowns showed better gingival health, less bleeding, and plaque accumulation, as well as less loss of material.
